# Prognostic impact of myelodysplasia-related gene mutations in ELN-2022 favorable-risk acute myeloid leukemia subtypes

**DOI:** 10.1080/07853890.2026.2636337

**Published:** 2026-03-09

**Authors:** Lulu Zhang, Shuangwei Ying, Fang Fang, Qian Li, Furun An, Jingwen Li, Jie Sun, Weiyan Zheng, Zhimin Zhai, Yuanyuan Zhu

**Affiliations:** a Department of Hematology, The Second Affiliated Hospital of Anhui Medical University, Hefei, China; b Department of Hematology, Taizhou Hospital of Zhejiang Province affiliated to Wenzhou Medical University, Linhai, China; c Department of Hematology, Tongling People’s Hospital, Tongling, China; d Bone Marrow Transplantation Center, The First Affiliated Hospital, Zhejiang University School of Medicine, Hangzhou, China

**Keywords:** Acute myeloid leukemia, AML, favorable-risk AML, myelodysplasia-related gene

## Abstract

**Background:**

The 2022 European Leukemia Net (ELN) risk stratification categorizes acute myeloid leukemia (AML) with myelodysplasia-related gene (MRG) mutations - including *ASXL1*, *BCOR*, *EZH2*, *RUNX1*, *SF3B1*, *SRSF2*, *STAG2*, *U2AF1* and/or *ZRSR2* - as “adverse-risk”. However, the prognostic relevance of MRG mutations in patients with favorable-risk AML remains uncertain.

**Methods:**

In this study, we analyzed a cohort of 221 adult patients with de novo favorable-risk AML. Risk groups were classified according to the 2022 European Leukemia Net guideline.

**Results:**

A total of 47 AML patients (21.3%) harbored MRG mutations. The presence of MRG mutations was associated with older age (57 vs. 49, *p* = 0.005), lower white blood cell count (6.9 vs. 14.5, *p* = 0.015), and the presence of *TET2* (27.7% vs. 10.9%, *p* = 0.004), *MPL* (6.4% vs. 0.6%, *p* = 0.031), and *ETV6* (6.4% vs. 1.1%, *p* = 0.066) mutations. Our findings indicated that the presence of MRG mutations did not significantly impact 2-year overall survival (OS) (75.2% vs. 69.4%, *p* = 0.285) or leukemia-free survival (LFS) (58.9% vs. 52.5%, *p* = 0.640). However, patients with two or more MRG mutations had significantly poorer LFS than those with one MRG mutation (*p* = 0.004) or without MRG mutations (*p* = 0.001). By multivariable analysis, ≥2 MRG mutations was independently associated with worse LFS.

**Conclusion:**

The presence of a single MRG mutation did not confer a worse prognosis in favorable-risk AML, whereas a high MRG mutation burden (≥2 mutations) was independently associated with poorer LFS. This study suggests that quantifying the MRG mutation burden may inform risk stratification in this patient population.

## Introduction

In 2022, a new acute myeloid leukemia (AML) entity was introduced in the classifications, defined by mutations in genes such as *ASXL1*, *BCOR*, *EZH2*, *RUNX1*, *SF3B1*, *SRSF2*, *STAG2*, *U2AF1*, and *ZRSR2*. Studies have established that this genetic signature is highly specific to secondary AML that evolves from a prior clonal hematopoiesis [1,2]. The International Consensus Classification (ICC) of Myeloid Neoplasms and Acute Leukemias [3] and the novel 5th edition of the World Health Organization (WHO) classification of myeloid neoplasms [4] further emphasized the importance of these markers. Patients harboring these mutations are classified as “AML with myelodysplasia-related gene (MRG) mutations” (ICC) and “AML, myelodysplasia-related” (AML-MR, WHO) respectively. These mutations are highly correlated with prior myelodysplastic syndromes (MDS) and lead to poor prognosis even if they occur in de novo AML [5,6].

The European Leukemia Network (ELN) updated the risk classification of acute myeloid leukemia (AML) In 2022 [7], building upon the framework originally introduced in 2010 [8]. This classification has become one of the most widely utilized frameworks for evaluating the prognosis of AML patients and guiding treatment choices. A major novelty of ELN 2022 is that MRG mutations were generally associated with adverse prognosis. The 2022 ELN risk classification considers patients with *CBFβ::MYH11*, *RUNX1::RUNX1T1*, mutated *NPM1* without *FLT3-ITD*, and in-frame mutations in the basic leucine zipper domain of *CEBPA* (*CEBPA*-bZip) as favorable risk irrespective of these mutations [7]. This raises an important question: does the presence of MRG mutations affect the prognosis of patients who otherwise meet the criteria for favorable-risk AML? The prognostic significance of MRG mutations in this specific patient subgroup remains uncertain, necessitating further investigation.

## Materials and methods

We performed a retrospective review of 221 patients aged ≥18 years with newly diagnosed AML, classified according to the 2016 World Health Organization (WHO) classification [3], who were treated at the Bone Marrow Transplantation Center of The First Affiliated Hospital, Zhejiang University School of Medicine between 2015 and 2023. All patients were categorized as favorable risk under the 2022 ELN guidelines. To ensure the rigorous and consistent application of these criteria, the risk stratification for each patient was systematically confirmed through an independent review of integrated cytogenetic and molecular genetic data by two senior hematologists (Supplementary Figure S1). Data were obtained from the patients’ medical records, including demographic information, laboratory test results, cytogenetic and molecular genetic data. DNA mutations were analyzed by next-generation sequencing (NGS) using an 87-gene panel associated with myeloid malignancies (Supplementary Table S1). Any patients who lacked NGS data at diagnosis were excluded from the study. The induction treatment was based on the physician’s assessment of patient fitness, age, and comorbidities. Intensive regimens included ‘7 + 3′ (cytarabine plus an anthracycline) or homoharringtonine combined with cytarabine and aclarubicin (HAA). Non-intensive regimens consisted of a low-dose cytarabine-based regime or venetoclax combined with azacytidine. Risk stratification and therapy response were defined based on the 2022 ELN guideline [7]. The study was approved by both institutional and national ethics committees. Due to its retrospective design, the requirement for informed consent was waived in accordance with institutional ethical guidelines and the Declaration of Helsinki. The last follow-up was censored on 31 August 2023.

Patient characteristics were summarized using medians and ranges for quantitative data, and counts and percentages for qualitative data. Continuous variables were expressed as means or medians and were analyzed using the Mann-Whitney U test, while categorical variables were compared using the Pearson chi-square test or Fisher’s exact test, as appropriate. Gene interactions were evaluated with pairwise Fisher’s exact tests, applying false discovery rate correction. Response to treatment was classified based on the 2022 ELN criteria, including complete remission (CR), CR with incomplete hematologic recovery (CRi), CR with partial hematologic recovery (CRh), and partial remission (PR). Overall survival (OS) was defined from diagnosis to death or the last follow-up. Leukemia-free survival (LFS) was defined as the time from the date of CR to the first relapse. Survival outcomes were analyzed using the Kaplan-Meier (KM) method, with comparisons made using the log-rank test. Multivariate regression analysis was performed using the Cox proportional hazards model to determine hazard ratios (HR) and 95% confidence intervals (CI). To assess the heterogeneity of the prognostic impact of MRG mutations across different genetic contexts, we performed subgroup analyses within each of the four favorable-risk subtypes. The interaction between MRG mutation status and genetic subtype was tested by including an interaction term in the Cox proportional hazards model, and the results are presented as forest plots. All statistical analyses and graphical representations were carried out using SPSS, version 22.0 (Chicago, IL, USA) and R software version 4.2.1. A two-sided P value <0.05 was considered statistically significant.

## Results

### Association with clinical characteristics

The demographic and hematologic features of the study population are summarized in Table 1. A total of 221 favorable-risk AML patients (100 male and 121 female) were included, with a median age at diagnosis of 50 years (range 18–81). MRG mutations (defined as the presence of one or more mutations in *ASXL1*, *BCOR*, *EZH2*, *SRSF2*, *ZRSR2*, *STAG2*, *SF3B1*, *RUNX1*, or *U2AF1*) were identified in 47 patients (21.3%).

**Table 1.** Baseline characteristics of patients with and without MRG mutations.

Characteristic	MRG-	MRG +	P
Number, n (%)	174 (78.7)	47 (21.3)	
Gender, n (%)			0.567
Male	77 (44.3)	23 (48.9)	
Female	97 (55.7)	24 (51.1)	
Age, years, median (range)	49 (18–72)	57 (21–81)	0.005
Bone marrow blasts %, median (range)	60 (15–97)	56 (22–90)	0.209
White blood cell count, ×10^9^/L, median (range)	14.5 (0.5–351.6)	6.9 (0.7–348.8)	0.015
Hemoglobin, g/L, median (range)	88 (28–157)	87 (47–132)	0.794
Platelet count, ×10^9^/L, median (range)	32 (3–292)	39 (2–220)	0.679
lactate dehydrogenase, IU/L, median (range)	464 (118–3133)	123 (123–1571)	0.123
FAB, n (%)			
M0	1 (0.6)	0 (0.0)	1.000
M1	9 (5.2)	2 (4.3)	1.000
M2	85 (48.9)	23 (48.9)	0.992
M4	22 (12.6)	5 (10.6)	0.710
M5	57 (32.8)	17 (36.2)	0.660
Gene, n (%)			
*CEBPA*-bZip	50 (28.7)	13 (27.7)	0.885
*CBFβ::MYH11*	40 (23.0)	5 (10.6)	0.062
*NPM1*	40 (23.0)	14 (29.8)	0.336
*RUNX1::RUNX1T1*	43 (24.7)	14 (29.8)	0.480
*CEBPA*-bZip and *NPM1*	1 (0.6)	1 (2.1)	0.897
Induction therapy, n (%)			
Intensive	112 (64.4)	14 (29.8)	<0.001
Non-Intensive	60 (34.5)	32 (68.1)	<0.001
Clinical trial	2 (1.1)	1 (2.1)	1.000
Allogeneic SCT, n (%)			0.332
No	105 (60.3)	32 (68.1)	
Yes	69 (39.7)	15 (31.9)	

Abbreviations: MRG mutations, *ASXL1*, *BCOR*, *EZH2*, *SRSF2*, *ZRSR2*, *STAG2*, *SF3B1*, *RUNX1*, and *U2AF1*; SCT, stem cell transplant.

To explore the impact of MRG mutations on favorable-risk AML, we compared the baseline characteristics between patients with and without MRG mutations. Patients with MRG mutations were significantly older (57 vs. 49, *p* = 0.005) and had lower white blood cell (WBC) counts at diagnosis (6.9 vs. 14.5, *p* = 0.015) compared to those without MRG mutations. MRG mutations were less frequent in AML with *CBFβ::MYH11* (10.6% vs. 23.0%, *p* = 0.062), with a trend toward significance. But there were no significant differences in sex, hemoglobin levels, platelet counts, lactate dehydrogenase levels, or bone marrow characteristics between the groups. In terms of treatment, fewer patients with MRG mutations received frontline intensive chemotherapy compared to those without MRG mutations (29.8% vs. 64.4%, *p* < 0.001), while a higher proportion of patients with MRG mutations received non-intensive regimens (68.1% vs. 34.5%, *p* < 0.001). The rates of stem cell transplantation (SCT) were comparable between the two groups (31.9% vs. 39.7%, *p* = 0.332).

### Association with genetic characteristics

Cytogenetic data were available for 167 patients, including 40 patients with MRG mutations and 127 patients without MRG mutations (Supplementary Table S2). The distribution of various karyotypic alterations was comparable between the two groups. This included similar frequencies of a normal karyotype (52.5% vs. 59.1%, *p* = 0.465), loss of sex chromosome (12.5% vs. 10.2%, *p* = 0.770), as well as low and comparable incidence of complex karyotype (2.5% vs. 0.8%, *p* = 0.423) and monosomal karyotype (2.5% vs. 2.4%, *p* = 1.000). Furthermore, classic MDS-associated cytogenetic abnormalities were absent or uncommon in patients with MRG mutations: −5/5q- was not observed, while −7/7q- and +8 were each present in only two patients (5.0%).

**Figure 1.**
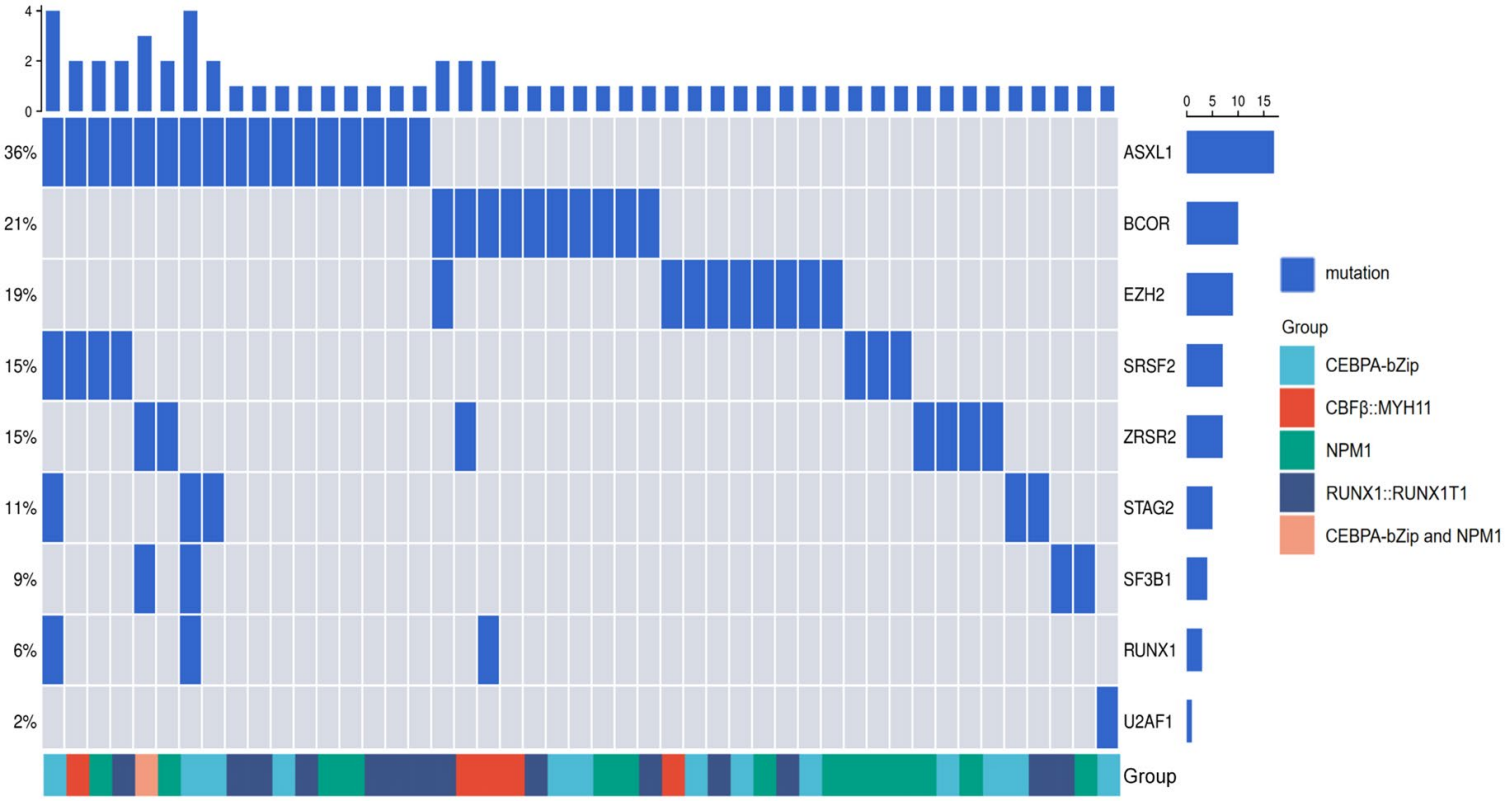
Mutational landscape in AML with MRG mutations. Each column represents an individual patient. The bar plot at the top illustrates the mutation burden for each case. The frequency of each mutation is displayed on the left, and the corresponding gene names are shown on the right.

**Figure 2.**
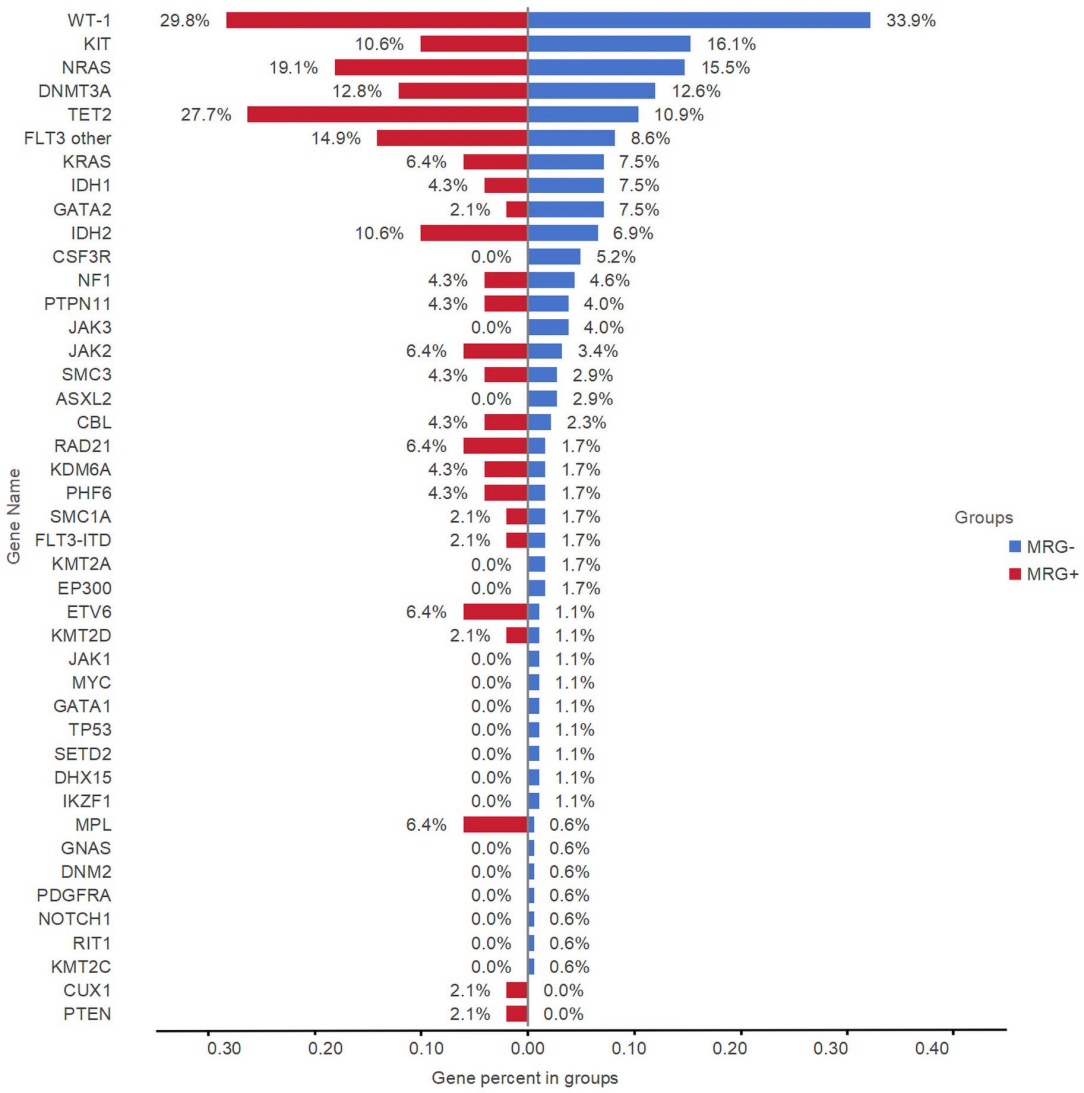
Differential distribution of co-occurring mutations in patients with and without MRG mutations.

Addressing the mutational spectrum of co-occurring mutations, all 47 patients with MRG mutations and 174 patients without MRG mutations underwent targeted NGS to assess AML-related gene mutations. Among patients with MRG mutations, the number of such mutations varied: 36 had one, 8 had two, 1 had three, and 2 had four mutations (Figure 1). The most frequently mutated MRG was *ASXL1* (17/47, 36.2%), followed by *BCOR* (10/47, 21.3%), *EZH2* (9/47, 19.1%), *ZRSR2* (7/47, 14.9%), *SRSF2* (7/47, 14.9%), *STAG2* (5/47, 10.6%), *SF3B1* (4/47, 8.5%), *RUNX1* (4/47, 8.5%) and *U2AF1* (1/47, 2.1%). Notably, *CEBPA*-bZip co-occurred with all MRG genes. Although MRG mutations were less common in the *CBFβ::MYH11* group, patients with *CBFβ::MYH11* showed a higher likelihood of having two or more MRG mutations compared to other favorable-risk subtypes (3/5, 60.0%). The average number of co-occurring mutations was comparable between patients with or without MRG mutations (2.3 vs. 2.0, range 0–7; *p* = 0.278). In both groups, the largest proportion of patients had exactly one additional mutation (29.8% vs. 28.2%; Supplementary Table S3). The overall mutational landscape differed significantly between the two groups (Figure 2; Supplementary Table S4). The most common co-occurring mutation was in Wilms-tumor1 (*WT1*) gene, with a comparable frequency between cohorts (29.8% vs. 33.9%, *p* = 0.594). The presence of MRG mutations was significantly associated with *TET2* mutations (27.7% vs. 10.9%, *p* = 0.004), which was the second most common co-occurring genetic aberration in the MRG-mutant cohort. Mutations in *MPL* (6.4% vs. 0.6%, *p* = 0.031) and *ETV6* (6.4% vs. 1.1%, *p* = 0.066) were also more frequent in the MRG-mutant group. Conversely, MRG mutations showed a trend toward mutual exclusivity with recurrent alterations in *CSF3R*, *JAK3*, *ASXL2* (*p* = 0.210, *p* = 0.350 and *p* = 0.587). The distribution of other studied mutations did not differ significantly between groups.

The detected mutations were categorized into different functional groups based on the functional classification of mutant genes (Supplementary Table S5). Among patients with MRG mutations, the most prevalent functional groups were chromatin modifiers (74.5%), transcription factors (66.0%), and activated signaling pathways (55.3%). Mutations in chromatin modifiers co-occurred more frequently with MRG mutations (74.5% vs. 8.0%, *p* < 0.001). Higher mutation rates in spliceosome (38.3% vs. 1.1%, *p* < 0.001), cohesin complex (21.3% vs. 5.7%, *p* = 0.003), DNA methylation pathways (46.8% vs. 30.5%, *p* = 0.036) were also observed in patients with MRG mutations. In patients without MRG mutations, transcription factors represented the most commonly mutated functional group (54.6%), followed by activated signaling pathways (46.6%) and tumor suppressors (35.6%). The mutation frequencies of these three functional groups were similarly distributed between patients with and without MRG mutations.

**Figure 3.**
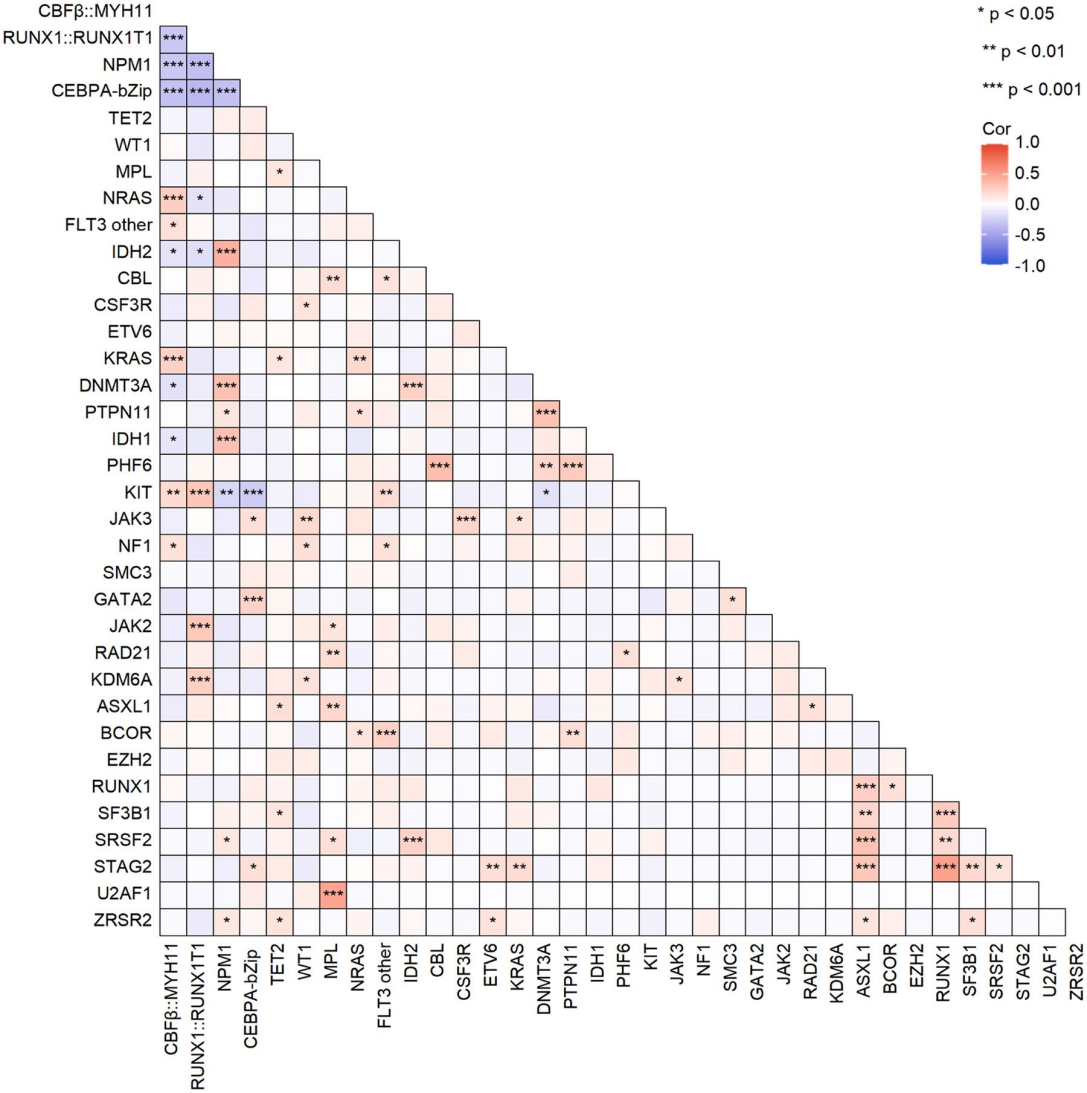
Gene interaction analysis included all genes mutated in >3% of patients (either with or without MRG mutations) and the *U2AF1* gene.

Certain mutations showed distinctive co-occurrence patterns. AML with *CBFβ::MYH11* fusion was more likely to harbor concurrent mutations in *NRAS*, *KRAS*, *KIT*, *FLT3* other (non-*ITD*), and *NF1*, but was negatively correlated with mutations in *IDH1*, *IDH2*, and *DNMT3A*. AML with *RUNX1::RUNX1T1* fusion frequently co-occurred with mutations in *KIT*, *JAK2*, and *KDM6A*, but was inversely related to mutations in *IDH2* and *NRAS. NPM1* mutations were commonly associated with *IDH1*, *IDH2*, *DNMT3A*, *ZRSR2* and *SRSF2. CEBPA*-bZip co-occurred with mutations in *STAG2*, *JAK3* and *GATA2*. Conversely, both *NPM1* and *CEBPA*-bZip were negatively correlated with *KIT* mutations. Mutations in *ASXL1* and *RUNX1* each exhibited a higher frequency of co-mutations with *SRSF2*, *SF3B1*, and *STAG2*. Other significant pairwise associations included *BCOR* and *FLT3* other, *SRSF2* and *IDH2*, as well as *U2AF1* and *MPL* (Figure 3).

### Impact of MRG mutations on response, outcome and survival

In this cohort, a higher proportion of patients without MRG mutations received frontline intensive chemotherapy and SCT, which is likely attributable to the younger age of this patient group relative to those with MRG mutations. Response to induction therapy was comparable between patients with and without MRG mutations (46.8% vs. 44.3% achieved CR, *p* = 0.755 and 29.8% vs. 35.6% achieved CRi/CRh, *p* = 0.454). However, patients with MRG mutations required more cycles of induction chemotherapy to achieve CR (8.9% vs. 1.8%, *p* = 0.037). The proportion of patients undergoing SCT during the first CR was similar between groups (23.4% vs. 27.0%, *p* = 0.768). Likewise, the number of patients who received SCT as salvage therapy was comparable (6.4% vs. 3.4%, *p* = 0.198). Finally, the frequencies of death (27.7% vs. 25.2%, *p* = 0.946) and relapse (34.0% vs. 40.2%, *p* = 0.440) were also comparable between the groups (Table 2).

**Table 2.** Response, outcome and SCT of in patients with and without MRG mutations.

		MRG- n (%)	MRG+ n (%)	P
Number		174 (78.7)	47 (21.3)	
SCT	no SCT	105 (60.3)	32 (68.1)	0.332
	SCT	69 (39.7)	15 (31.9)	
	SCT CR1	47 (27.0)	11 (23.4)	0.768
	SCT CR2	16 (9.2)	1 (2.1)	0.285
	SCT NR	6 (3.4)	3 (6.4)	0.198
Outcome	Death	49 (28.2)	13 (27.7)	0.946
	Events	85 (48.9)	18 (38.3)	0.198
	Relapse	70 (40.2)	16 (34.0)	0.440
Induction responses	CR	77 (44.3)	22 (46.8)	0.755
	CRi/CRh	62 (35.6)	14 (29.8)	0.454
	PR/Refractory	34 (19.5)	11 (23.4)	0.559
	Induction death	1 (0.6)	0 (0.0)	1.000
Induction cycle	1 course	140 (82.8)	36 (80.0)	0.658
	2 course	26 (15.4)	5 (11.1)	0.469
	>2 course	3 (1.8)	4 (8.9)	0.037

Abbreviations: MRG mutations, *ASXL1*, *BCOR*, *EZH2*, *SRSF2*, *ZRSR2*, *STAG2*, *SF3B1*, *RUNX1*, and *U2AF1*; SCT, stem cell transplant; CR, complete remission; CRi, CR with incomplete hematologic recovery; CRh, CR with partial hematologic recovery; PR, partial remission; NR, non-remission.

**Figure 4.**
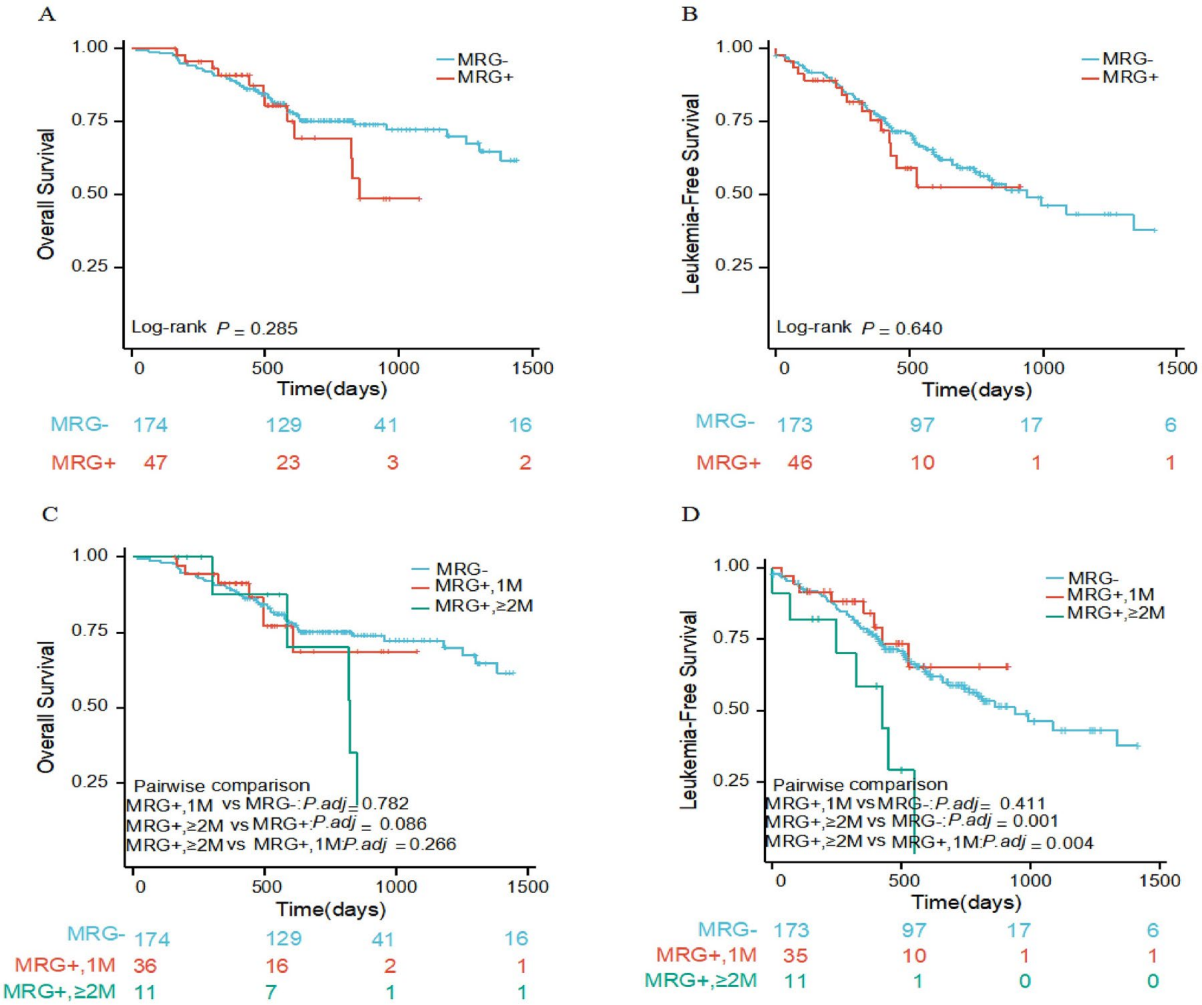
The Kaplan–Meier survival curves for the cohort. (A) OS and (B) LFS show no significant differences between patients with and without MRG mutations. (C) OS and (D) LFS stratified by MRG mutation burden. Median LFS was significantly shorter in patients with two or more MRG mutations compared to those with a single or no MRG mutation.

The median follow-up for the entire cohort was 25.8 ± 1.0 months (95% CI, 23.8–27.8), and the 2-year LFS and OS in the entire cohort were 57.7% and 74.6%, respectively. The presence of MRG mutations had no significant effect on 2-year OS (75.2% vs. 69.4%, *p* = 0.285) and LFS (58.9% vs. 52.5%, *p* = 0.640) (Figure 4A, B). We further stratified patients by the number of MRG mutations. Patients with two or more MRG mutations had significantly poorer LFS than those with only a single MRG mutation (*p* = 0.004) or without MRG mutations (*p* = 0.001). However, no significant difference in OS was observed based on the number of MRG mutations. Moreover, patients with only one MRG mutation showed comparable OS (*p* = 0.782) and LFS (*p* = 0.411) to those without MRG mutations (Figure 4C, D).

The complete remission rates did not differ significantly among patients with *NPM1*-mutated, *CEBPA*-mutated, *CBFβ::MYH11*, and *RUNX1::RUNX1T1*, regardless of the presence of additional MRG mutations (Supplementary Table S6). In the subgroup analysis of patients with *CEBPA* mutations, those who harbored MRG mutations (*n* = 13) had significantly worse OS than those without MRG mutations (*n* = 50) (19.5 months vs. NR, *p* = 0.005), and MRG mutations showed a trend towards shorter LFS (11.8 months vs. 22.0 months, *p* = 0.131). Among patients with *CBFβ::MYH11*, MRG mutations were associated with significantly adverse LFS (15.0 months vs. 44.5 months, *p* = 0.001) but not OS (NR vs. NR, *p* = 0.600). In contrast, MRG mutations did not significantly affect LFS or OS in patients with *NPM1*-mutated AML or *RUNX1::RUNX1T1* AML. To determine whether the prognostic effect of MRG mutations was consistent across different genetic contexts, we tested for heterogeneity across the four favorable-risk genetic subtypes. The tests for interaction revealed that the impact of MRG mutations on both OS and LFS was significantly dependent on the genetic subtype (P for interaction for OS = 0.025; P for interaction for LFS = 0.021). The corresponding Kaplan-Meier survival curves and forest plots for each subtype are provided in Supplementary Figure S2 and S3, respectively. However, the limited number of patients in some of these genetic subgroups necessitates caution in interpreting these findings, and they require validation in larger cohorts.

### Prognostic factors in Favorable-risk AML subtypes

Univariate analysis demonstrated that age ≥50 years at diagnosis, *KIT* mutation, and non-intensive frontline treatment (compared to intensive treatment) were significantly associated with worse OS. Age ≥50 years, platelet < 34 × 10^9^/L, *KIT* mutation and the presence of ≥2 MRG mutations (compared to either one or no MRG mutations) were negatively associated with LFS. Patients who underwent SCT had a significantly better OS than those who did not. There was no significant difference in LFS between patients who underwent SCT and those who did not. Multivariable analysis confirmed that *KIT* mutation remained an independent prognostic factor for both OS and LFS. Furthermore, the presence of ≥2 MRG mutations remained independently associated with worse LFS (Table 3).

**Table 3.** Multivariable analyses for factors associated with leukemia-free survival and overall survival.

	Univariate			Multivariate		
Variable	HR	95%CI	P	HR	95%CI	P
**Overall Survival**						
^a^MRG mutations						
Mutation burden =1	1.13	0.53–2.42	0.751	0.96	0.435–2.11	0.915
Mutation burden ≧2	2.21	0.88–5.56	0.093	1.50	0.58–3.85	0.403
Age at diagnosis	2.84	1.64–4.92	<0.001	2.22	1.24–3.98	0.008
platelet < 34	0.72	0.43–1.21	0.212	0.64	0.38–1.08	0.097
*KIT* mutation	2.29	1.28–4.11	0.006	2.37	1.28–4.38	0.006
intensive frontline treatment	0.56	0.34–0.92	0.022	0.60	0.36–0.98	0.042
Allogeneic SCT	0.56	0.32–0.97	0.038	0.62	0.34–1.12	0.105
**Leukemia-Free Survival**						
^a^MRG mutations						
Mutation burden =1	0.75	0.37–1.51	0.423	0.69	0.34–1.42	0.316
Mutation burden ≧2	3.42	1.54–7.57	0.002	3.04	1.33–6.92	0.008
Age at diagnosis	1.72	1.13–2.61	0.011	1.52	0.97–2.40	0.069
platelet < 34	0.47	0.30–0.73	0.001	0.45	0.29–0.71	0.001
*KIT* mutation	1.97	1.18–3.29	0.009	1.80	1.01–3.10	0.034
intensive frontline treatment	0.75	0.49–1.14	0.175	0.71	0.46–1.11	0.135
Allogeneic SCT	0.92	0.60–1.41	0.691	0.99	0.62–1.57	0.960

Abbreviations: MRG mutations, *ASXL1*, *BCOR*, *EZH2*, *SRSF2*, *ZRSR2*, *STAG2*, *SF3B1*, *RUNX1*, and *U2AF1*; SCT, stem cell transplant; CI, Confidence interval; HR, hazard ratio.

^a^Reference level is No MRG mutations.

## Discussion

In the ELN 2017 guidelines, only *ASXL1* and *RUNX1* were considered adverse-risk for AML in the absence of concurrent favorable-risk subtypes [9]. The mutational signature now known as MRG mutations has been reported to be highly specific to secondary AML. The decision to include MRG mutations as prognostic markers was based on studies providing compelling evidence that linked MRG mutations to a poor prognosis in de novo AML patients [1,5,6]. Based on this, the ELN 2022 version integrated the latest developments regarding the prognostic significance of AML genetic alterations and added seven mutations associated with myelodysplastic syndromes: *BCOR*, *EZH2*, *SF3B1*, *SRSF2*, *STAG2*, *U2AF1*, and *ZRSR2*. However, these markers should not currently be considered adverse prognostic marker if they co-occur with favorable-risk AML subtypes, need to be further confirmed [7].

This is the first real-world study to explore the prognostic significance of MRG mutations in favorable-risk AML as defined by the 2022 ELN criteria. Preliminary findings from this cohort were previously presented in abstract form [10]. In this retrospective cohort of 221 newly diagnosed AML patients. In this cohort, we observed that MRG mutations occur in a high incidence up to 21.3% and the majority of patients carry only a single MRG mutation. Previous studies primarily focused on NPM1-mutated AML, reporting incidences of MRG mutations ranging from 13% to 19%, particularly in older patients with lower WBC and platelet counts [11–13]. Consistent with these findings, our study also found that MRG mutations were more frequent in older patients and in those with a lower WBC count at diagnosis. Although the incidence of MRG mutations was low in patients with *CBFβ::MYH11* fusion, their presence was associated with significantly poorer LFS, potentially due to a higher frequency of two or more MRG mutations in this subgroup.

We analyzed the mutational profiles of patients with and without MRG mutations. *ASXL1* was the most frequently mutated MRG in this cohort. Among the splicing factor mutations (*ZRSR2*, *SRSF2*, *SF3B1*, *U2AF1*), *SRSF2* and *ZRSR2* mutations were more common in *NPM1*-mutated AML (4/7, 57%). Previous studies have reported a high incidence of *SRSF2* mutations (38-66%) in *NPM1*-mutated AML patients with concurrent MRG mutations [11,13,14]. *WT1* was the most common co-occurring mutation in both groups. *TET2* was also a frequent co-occurring mutation in patients with MRG mutations and was often found alongside *ZRSR2*, *SRSF2*, and *ASXL1*, suggesting a potential cooperative interaction between these mutations. *TET2* is seen in approximately 10% of de novo AML and 30% of MDS cases, and is associated with DNA hypermethylation, increased risk of MDS progression and poor prognosis in AML [15]. In our study, the frequency of *TET2* mutations in patients with MRG mutations was similar to that observed in MDS; however, it did not show a significant prognostic impact in this context.

Previous studies have categorized gene mutations based on the functional pathways of the altered genes [6,16,17]. Based on this framework, our analysis revealed higher incidences of mutations in the spliceosome, chromatin modifiers, and cohesin complex in patients with MRG mutations. Concurrent genetic aberrations were more frequent in patients with MRG mutations, particularly among genes involved in DNA methylation, with a specific propensity toward *TET2*. Among cohesin complex genes, *RAD21* showed a specific association with MRG mutations, whereas other cohesin complex-related aberrations were comparably distributed between groups. No significant differences were observed in tumor suppressor genes; however, transcription factors and activated signaling pathways tended to be more frequently altered in cases with MRG mutations, indicating a distinct genetic profile.

In this study, the presence of MRG mutations did not show prognostic significance for outcomes and survival in the general favorable-risk AML population. Although patients with MRG mutations required more induction chemotherapy cycles to achieve remission, this did not translate into an adverse overall prognosis. In this context, the interpretation of comparable survival should take into account that these patients more frequently received less intensive therapies-a factor potentially influencing outcomes through minimized treatment-related toxicity and preserved quality of life. Further subgroup analysis revealed that MRG mutations had no impact on the remission rates across the four of favorable-risk AML subtypes. However, in *CEBPA*-mutated patients, MRG mutations were associated with significantly poorer OS. Although there was no statistically significant difference in LFS, MRG mutations demonstrated a trend toward worse outcomes. In patients with *CBFβ::MYH11*, MRG mutations were associated with poor LFS but did not show a significant impact on OS. This suggests that MRG mutations may play an important role in disease progression. Previous studies on the impact of MRG mutations in *NPM1*-mutated patients have yielded inconsistent results [11–13]. The largest retrospective study indicated that MRG mutations had no effect on CR, OS and RFS, suggesting that they did not adversely affect clinical outcomes in *NPM1*-mutated patients, a finding that aligns with our results. Together, these findings reveal significant heterogeneity in the prognostic impact of MRG mutations across genetic subtypes, highlighting that their biological and clinical consequences are not uniform but are critically shaped by the coexisting molecular context. However, the limited number of patients in certain analyzed subgroups limits the current analysis, and further validation is needed to confirm these observations. Furthermore, the sample size, particularly within these subgroups, reduces the statistical power of our analyses. Additionally, as our study was confined to the favorable-risk population per ELN-2022 criteria, the generalizability of our findings to intermediate- or adverse-risk AML patients cannot be extrapolated from the present dataset. Therefore, the results, especially from subgroup comparisons, should be interpreted with caution and are best considered hypothesis-generating. Collectively, these findings suggest that the impact of MRG mutations on prognosis varies based on the molecular context, emphasizing the importance of genotype-guided personalized treatment approaches in the effective management of leukemia.

Interestingly, while MRG mutations did not independently predict poor OS or LFS, a higher MRG mutation burden (≥2 mutations) was significantly associated with worse LFS. This observation is supported by prior studies in which the presence of ≥2 MRG gene mutations was identified as a defining feature of a distinct, high-risk molecular class in AML, uniformly associated with an adverse prognosis [18]. While a similar trend was noted in an *NPM1*-mutated cohort, it did not reach significance, likely due to the limited number of such patients [19]. These findings provide further support for the ‘mutation burden’ concept, demonstrating its relevance even within a favorable-risk context and suggesting that the cumulative effect of multiple MRG mutations may be a better reflector of the actual prognosis than the presence of any single MRG mutation. Specifically, patients with two or more MRG mutations had significantly inferior LFS, indicating that the combined effect of multiple mutations may drive disease progression. This finding highlights the necessity to reassess how favorable-risk genetic subtypes are interpreted in the context of co-occurring MRG mutations, as the current 2022 ELN guidelines may not fully incorporate the prognostic implications of mutation burden. However, it is critical to emphasize that this analysis included only 11 patients with ≥2 MRG mutations. It should also be noted that, although treatment intensity was adjusted for in the multivariable model, its non-randomized assignment remains a potential residual confounder for this specific finding. Finally, the lack of centralized morphological review limits any conclusions regarding dysplasia-defined AML-MR, and our analysis focuses on the molecular MRG status. Therefore, this finding must be considered exploratory and hypothesis-generating, requiring validation in larger, independent cohorts before any clinical implications can be drawn.

In conclusion, this study provides novel insights into the role of MRG mutations in favorable-risk AML and suggests the critical importance of considering both the number and interactions of mutations in risk stratification. Although a single MRG mutation did not significantly impact overall survival or LFS, the presence of multiple MRG mutations was associated with a poorer prognosis, highlighting the need for a more refined approach to risk stratification in AML. Future research should involve larger, multi-center cohorts to validate these findings and optimize individualized treatment strategies for AML patients.

## Supplementary Material

Figure and Table Legends.docx

IANN-2025-5157.R2-Supplementart Table -Clean copy.docx

Revised_Manuscript _Clean.docx

## Data Availability

The datasets utilized and analyzed in this study are available from the corresponding author upon reasonable request.
