# Supplementary material for: Prognostic impact of myelodysplasia-related gene mutations in ELN-2022 favorable-risk acute myeloid leukemia subtypes
Source: Ann Med. 2026 Mar 9;58(1):2636337. doi: 10.1080/07853890.2026.2636337 (PMC12973831; doi:10.1080/07853890.2026.2636337)
Supplement: Figure and Table Legends.docx [file IANN_A_2636337_SM1570.docx]

**Table 1.** Baseline characteristics of patients with and without MRG mutations.

Abbreviations: MRG mutations, *ASXL1*, *BCOR*, *EZH2*, *SRSF2*, *ZRSR2*, *STAG2*, *SF3B1*, *RUNX1*, and *U2AF1*; SCT, stem cell transplant.

**Table 2.** Response, outcome and SCT of in patients with and without MRG mutations.

Abbreviations: MRG mutations, *ASXL1*, *BCOR*, *EZH2*, *SRSF2*, *ZRSR2*, *STAG2*, *SF3B1*, *RUNX1*, and *U2AF1*; SCT, stem cell transplant; CR, complete remission; CRi, CR with incomplete hematologic recovery; CRh, CR with partial hematologic recovery; PR, partial remission; NR, non-remission.

**Table 3.** Multivariable analyses for factors associated with leukemia-free survival and overall survival

Abbreviations: MRG mutations, *ASXL1*, *BCOR*, *EZH2*, *SRSF2*, *ZRSR2*, *STAG2*, *SF3B1*, *RUNX1*, and *U2AF1*; SCT, stem cell transplant; CI, Confidence interval; HR, hazard ratio.

^a^Reference level is No MRG mutations

**Figure 1.** Mutational landscape in AML with MRG mutations. Each column represents an individual patient. The bar plot at the top illustrates the mutation burden for each case. The frequency of each mutation is displayed on the left, and the corresponding gene names are shown on the right.

**Figure 2.** Differential distribution of co-occurring mutations in patients with and without MRG mutations.

**Figure 3.** Gene interaction analysis included all genes mutated in >3% of patients (either with or without MRG mutations) and the *U2AF1* gene.

**Figure 4.** The Kaplan‒Meier survival curves for the cohort. (A) OS and (B) LFS show no significant differences between patients with and without MRG mutations. (C) OS and (D) LFS stratified by MRG mutation burden. Median LFS was significantly shorter in patients with two or more MRG mutations compared to those with a single or no MRG mutation.

**Supplementary Table S1** Panel for next-generation sequencing.

**Supplementary Table S2** Cytogenetic aberrations in MRG+ and MRG- cohorts.

Abbreviations: KT, karyotype

**Supplementary Table S3** Number of molecular genetic mutations detected by NGS in MRG+ and MRG- cohorts.

**Supplementary Table S4.** Mutational status of leukemia associated genes analyzed by NGS in MRG+ and MRG- cohorts.

**Supplementary Table S5** Functional group of genetic mutation. Distribution of mutated genetic groups in MRG+ and MRG- cohorts.

**Supplementary Table S6** Summary of patient outcome with respect to MRG+ in four favorable-risk AML subtypes.

Abbreviations: CR, complete remission; CRi, CR with incomplete hematologic recovery; CRh, CR with partial hematologic recovery; OS, overall survival; LFS, leukemia-free survival; NR, median survival not reached.

Square brackets show 95%-confidence intervals.

**Supplementary Figure S1.** Flowchart of patient selection in the retrospective study.

**Supplementary Figure S2.**Kaplan-Meier survival analysis of favorable-risk AML genetic subtypes according to MRG mutation status. (A) OS and (B) LFS in patients with *CEBPA-bZip* mutations. (C) OS and (D) LFS in patients with *CBFB::MYH11* fusion. (E) OS and (F) LFS in patients with *NPM1* mutations. (G) OS and (H) LFS in patients with *RUNX1::RUNX1T1* fusion.

**Supplementary Figure S3.** Heterogeneity of the prognostic impact of MRG mutations on overall survival (OS) and leukemia-free survival (LFS). Forest plots display the hazard ratios (HR) for OS (left) and LFS (right) comparing patients with and without MRG mutations within each of the four favorable-risk subtypes. Tests for interaction revealed significant heterogeneity in the effect of MRG mutations across subtypes for OS and LFS (P for interaction for OS = 0.025; P for interaction for LFS = 0.021).
