# Supplementary material for: Prognostic impact of myelodysplasia-related gene mutations in ELN-2022 favorable-risk acute myeloid leukemia subtypes
Source: Ann Med. 2026 Mar 9;58(1):2636337. doi: 10.1080/07853890.2026.2636337 (PMC12973831; doi:10.1080/07853890.2026.2636337)

**Supplementary Information**

**Supplementary Table S1** Panel for next-generation sequencing.

| *ANKRD26* | *CDKN1B* | *EFL1* | *IDH1* | *KIT* | *NPM1* | *PTPN11* | *SOS1* | *TERT* |
| --- | --- | --- | --- | --- | --- | --- | --- | --- |
| *ASXL1* | *CEBPA* | *ELANE* | *IDH2* | *KMT2A* | *NRAS* | *RAD21* | *SPI1* | *TET2* |
| *ASXL2* | *CSF3R* | *EP300* | *IKZF1* | *KMT2C* | *NT5C2* | *RIT1* | *SRCAP* | *TP53* |
| *BCL11B* | *CTCF* | *ETV6* | *IKZF2* | *KMT2D* | *PAX5* | *RUNX1* | *SRP72* | *U2AF1* |
| *BCOR* | *CUX1* | *EZH2* | *IKZF3* | *KRAS* | *PDGFRA* | *SBDS* | *SRSF2* | *WT1* |
| *BCORL1* | *DDX41* | *FBXW7* | *JAK1* | *MPL* | *PDGFRB* | *SETBP1* | *STAG1* | *ZEB2* |
| *BRAF* | *DHX15* | *FLT3* | *JAK2* | *MSH6* | *PHF6* | *SETD2* | *STAG2* | *ZRSR2* |
| *CALR* | *DIS3* | *GATA1* | *JAK3* | *MYC* | *PPM1D* | *SF3B1* | *STAT3* |  |
| *CBL* | *DNM2* | *GATA2* | *KDM5C* | *NF1* | *PRPF8* | *SMC1A* | *SUZ12* |  |
| *CCND3* | *DNMT3A* | *GNAS* | *KDM6A* | *NOTCH1* | *PTEN* | *SMC3* | *TERC* |  |

**Supplementary Table S2** Cytogenetic aberrations in MRG+ and MRG- cohorts.

|  | **MRG-**  **(n=127, %)** | **MRG+**  **(n=40, %)** | **P** |
| --- | --- | --- | --- |
| Normal KT | 75 (59.1) | 21 (52.5) | 0.465 |
| Complex KT | 1 (0.8) | 1 (2.5) | 0.423 |
| Monosomal KT | 3 (2.4) | 1 (2.5) | 1.000 |
| t(8;21) | 30 (23.6) | 10 (25.0) | 0.859 |
| inv16/t(16;16) | 4 (3.1) | 2 (5.0) | 0.630 |
| -3/3q | 2 (1.6) | 0 (0.0) | 1.000 |
| -7/7q | 2 (1.6) | 2 (5.0) | 0.243 |
| -9/9q | 4 (3.1) | 2 (5.0) | 0.630 |
| -X/Y | 13 (10.2) | 5 (12.5) | 0.770 |
| -11 | 3 (2.4) | 0 (0.0) | 1.000 |
| -15 | 2 (1.6) | 0 (0.0) | 1.000 |
| +8 | 3 (2.4) | 2 (5.0) | 0.594 |
| +21 | 2 (1.6) | 0 (0.0) | 1.000 |
| +22 | 3 (2.4) | 1 (2.5) | 1.000 |
| t (9;22) | 1 (0.8) | 1 (2.5) | 0.423 |
| t (9;11) | 1 (0.8) | 1 (2.5) | 0.423 |

Abbreviations: KT, karyotype

**Supplementary Table S3** Number of molecular genetic mutations detected by NGS in MRG+ and MRG- cohorts.

| **number of additional mutations** | **MRG-**  **n (%)** | **MRG+**  **n (%)** | **P** |
| --- | --- | --- | --- |
| 0 | 34 (19.5) | 5 (10.6) | 0.155 |
| 1 | 50 (28.7) | 17 (36.2) | 0.325 |
| 2 | 34 (19.5) | 9 (19.1) | 0.952 |
| 3 | 31 (17.8) | 11 (23.4) | 0.386 |
| 4 | 20 (11.5) | 1 (2.1) | 0.053 |
| ≥5 | 5 (2.9) | 4 (8.5) | 0.099 |
| Median | 2.0 | 2.0 | 0.551 |
| Range | 0-5 | 0-6 |  |

**Supplementary Table S4.** Mutational status of leukemia associated genes analyzed by NGS in MRG+ and MRG- cohorts.

| **NGS data** | **MRG-**  **n (%)** | **MRG+**  **n** **(%)** | **P** |
| --- | --- | --- | --- |
| *WT-1* | 59 (33.9) | 14 (29.8) | 0.594 |
| *KIT* | 28 (16.1) | 5 (10.6) | 0.352 |
| *NRAS* | 27 (15.5) | 9 (19.1) | 0.550 |
| *DNMT3A* | 22 (12.6) | 6 (12.8) | 1.000 |
| *TET2* | 19 (10.9) | 13 (27.7) | 0.004 |
| *FLT3* | 15 (8.6) | 7 (14.9) | 0.269 |
| *KRAS* | 13 (7.5) | 3 (6.4) | 1.000 |
| *IDH1* | 13 (7.5) | 2 (4.3) | 0.743 |
| *GATA2* | 13 (7.5) | 1 (2.1) | 0.311 |
| *IDH2* | 12 (6.9) | 5 (10.6) | 0.368 |
| *CSF3R* | 9 (5.2) | 0 (0.0) | 0.210 |
| *NF1* | 8 (4.6) | 2 (4.3) | 1.000 |
| *PTPN11* | 7 (4.0) | 2 (4.3) | 1.000 |
| *JAK3* | 7 (4.0) | 0 (0.0) | 0.350 |
| *JAK2* | 6 (3.4) | 3 (6.4) | 0.405 |
| *SMC3* | 5 (2.9) | 2 (4.3) | 0.642 |
| *ASXL2* | 5 (2.9) | 0 (0.0) | 0.587 |
| *CBL* | 4 (2.3) | 2 (4.3) | 0.610 |
| *RAD21* | 3 (1.7) | 3 (6.4) | 0.112 |
| *KDM6A* | 3 (1.7) | 2 (4.3) | 0.288 |
| *PHF6* | 3 (1.7) | 2 (4.3) | 0.288 |
| *FLT3-ITD* | 3 (1.7) | 1 (2.1) | 1.000 |
| *KMT2A* | 3 (1.7) | 0 (0.0) | 1.000 |
| *EP300* | 3 (1.7) | 0 (0.0) | 1.000 |
| *SMC1A* | 3 (1.7) | 1 (2.1) | 1.000 |
| *ETV6* | 2 (1.1) | 3 (6.4) | 0.066 |
| *KMT2D* | 2 (1.1) | 1 (2.1) | 0.514 |
| *JAK1* | 2 (1.1) | 0 (0.0) | 1.000 |
| *MYC* | 2 (1.1) | 0 (0.0) | 1.000 |
| *GATA1* | 2 (1.1) | 0 (0.0) | 1.000 |
| *TP53* | 2 (1.1) | 0 (0.0) | 1.000 |
| *SETD2* | 2 (1.1) | 0 (0.0) | 1.000 |
| *DHX15* | 2 (1.1) | 0 (0.0) | 1.000 |
| *IKZF1* | 2 (1.1) | 0 (0.0) | 1.000 |
| *MPL* | 1 (0.6) | 3 (6.4) | 0.031 |
| *GNAS* | 1 (0.6) | 0 (0.0) | 1.000 |
| *DNM2* | 1 (0.6) | 0 (0.0) | 1.000 |
| *PDGFRA* | 1 (0.6) | 0 (0.0) | 1.000 |
| *RIT1* | 1 (0.6) | 0 (0.0) | 1.000 |
| *KMT2C* | 1 (0.6) | 0 (0.0) | 1.000 |
| *CUX1* | 0 (0.0) | 1 (2.1) | 0.213 |
| *PTEN* | 0 (0.0) | 1 (2.1) | 0.213 |
| no mutation | 34(19.5) | 5 (10.6) | 0.155 |

**Supplementary Table S5** Functional group of genetic mutation. Distribution of mutated genetic groups in MRG+ and MRG- cohorts.

| **functional group of additional mutation** | **MRG-**  **n (%)** | **MRG+**  **n (%)** | **P** |
| --- | --- | --- | --- |
| Spliceosome | 2 (1.1) | 18 (38.3) | ＜0.001 |
| Chromatin modifier | 14 (8.0) | 35 (74.5) | ＜0.001 |
| Tumor suppressor | 62 (35.6) | 15 (32.0) | 0.635 |
| Transcription factors | 95 (54.6) | 31 (66.0) | 0.163 |
| Cohesin complex | 10 (5.7) | 10 (21.3) | 0.003 |
| DNA methylation | 53 (30.5) | 22 (46.8) | 0.036 |
| Activated signaling | 81 (46.6) | 26 (55.3) | 0.286 |

**Supplementary Table S6** Summary of patient outcome with respect to MRG+ in four Favorable-risk AML Subtypes.

| **Outcome** | **MRG-** | **MRG+** | **P value** |
| --- | --- | --- | --- |
| *CEBPA-bZip* |  |  |  |
| Number, n (%) | 50 (79.4 ) | 13 (20.6 ) |  |
| CR/CRi/CRh, n (%) | 43 (86.0 ) | 9 (69.2 ) | 0.216 |
| Median LFS, months | 22.0 [15.3-28.7] | 11.8 [3.7-19.9] | 0.131 |
| Median OS, months | NR | 19.5 [14.7-24.3] | 0.005 |
| *CBFβ::MYH11* |  |  |  |
| Number, n (%) | 40 (88.9 ) | 5 (11.1 ) |  |
| CR/CRi/CRh, n (%) | 35 (87.5 ) | 5 (100 ) | 1.000 |
| Median LFS, months | 44.5 [21.1-67.9] | 15.0 [13.3-16.7] | 0.001 |
| Median OS, months | NR | NR | 0.600 |
| *NPM1* |  |  |  |
| Number, n (%) | 40 (74.1) | 14 ( 25.9) |  |
| CR/CRi/CRh, n (%) | 27 (67.5) | 10 (71.4) | 1.000 |
| Median LFS, months | NR | NR | 0.389 |
| Median OS, months | 50.2 [40.4-60.0] | NR | 0.191 |
| *RUNX1::RUNX1T1* |  |  |  |
| Number, n (%) | 43 (75.4) | 14 ( 24.6) |  |
| CR/CRi/CRh, n (%) | 34 (79.1) | 11 (78.9) | 1.000 |
| Median LFS, months | 20.3 [4.4-36.3] | NR | 0.175 |
| Median OS, months | NR | NR | 0.505 |

Abbreviations: CR, complete remission; CRi, CR with incomplete hematologic recovery; CRh, CR with partial hematologic recovery; OS, overall survival; LFS, leukemia-free survival; NR, median survival not reached.

Square brackets show 95%-confidence intervals.

**Supplementary Figure S1.** Flowchart of patient selection in the retrospective study


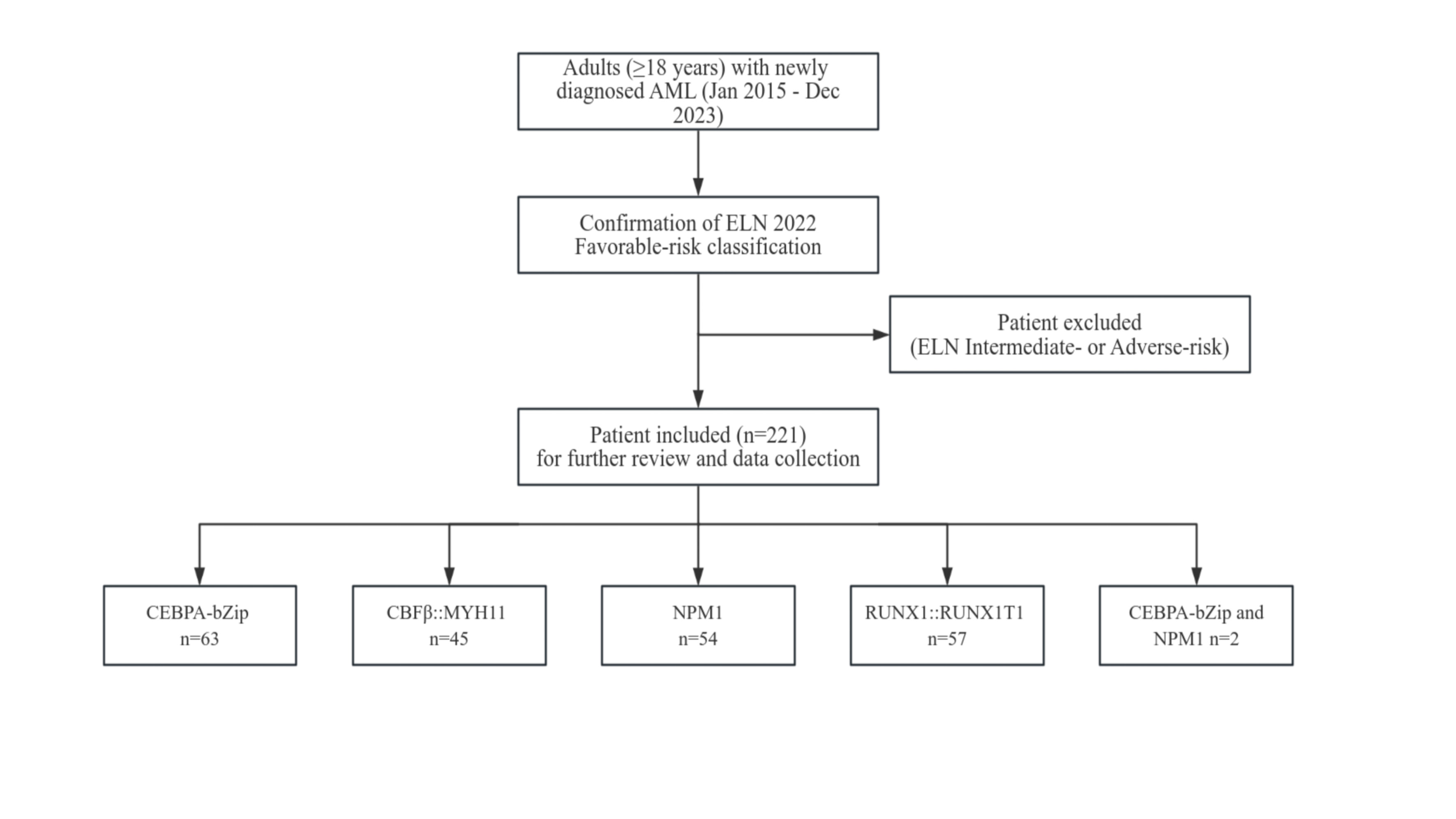


**Supplementary Figure S2.**Kaplan-Meier survival analysis of favorable-risk AML genetic subtypes according to MRG mutation status. (A) OS and (B) LFS in patients with *CEBPA-bZip* mutations. (C) OS and (D) LFS in patients with *CBFB::MYH11* fusion. (E) OS and (F) LFS in patients with *NPM1* mutations. (G) OS and (H) LFS in patients with *RUNX1::RUNX1T1* fusion.

**
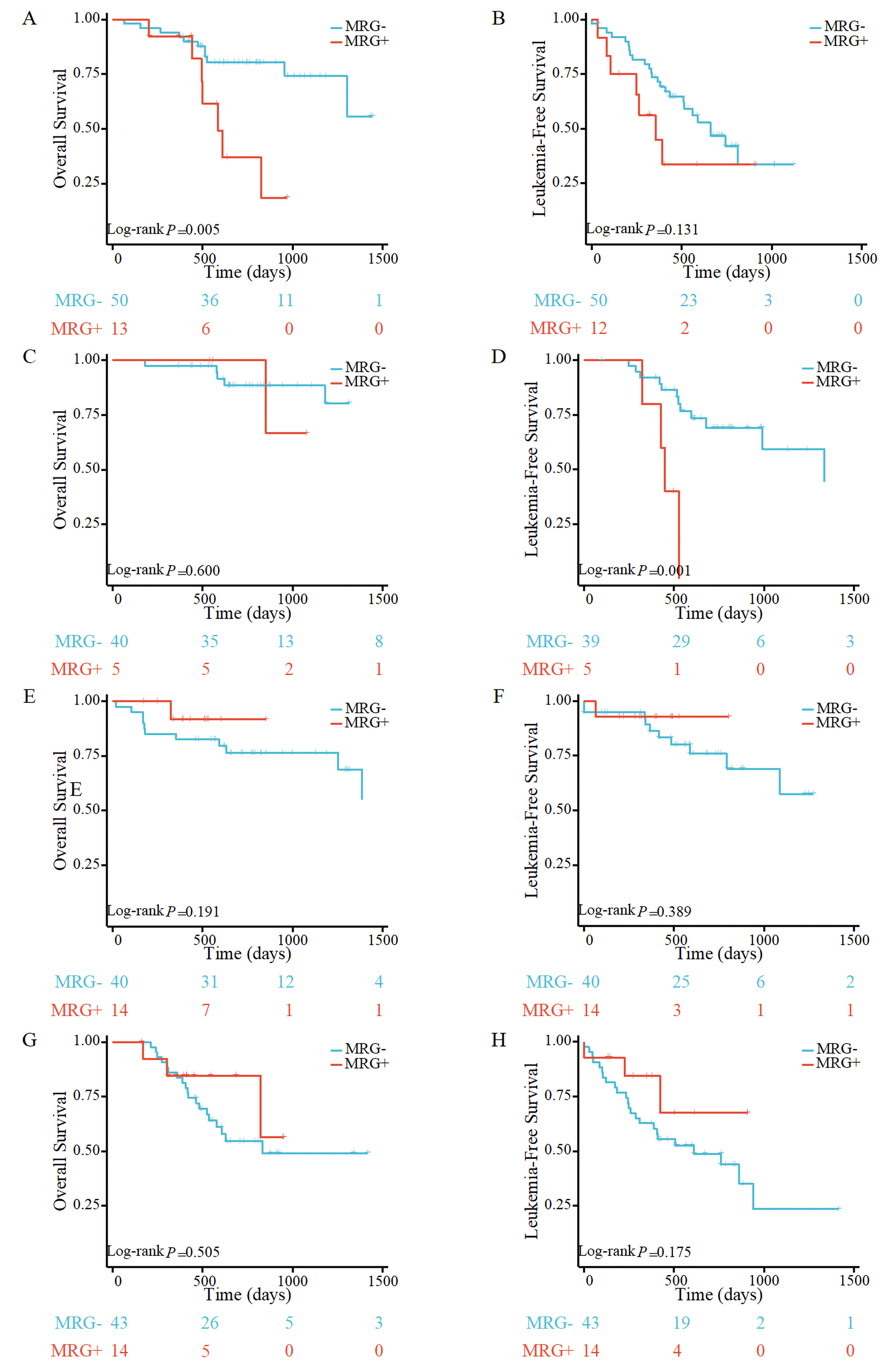
**

**Supplementary Figure S3.** Heterogeneity of the prognostic impact of MRG mutations on overall survival (OS) and leukemia-free survival (LFS). Forest plots display the hazard ratios (HR) for OS (left) and LFS (right) comparing patients with and without MRG mutations within each of the four favorable-risk subtypes. Tests for interaction revealed significant heterogeneity in the effect of MRG mutations across subtypes for OS and LFS (P for interaction for OS = 0.025; P for interaction for LFS = 0.021).


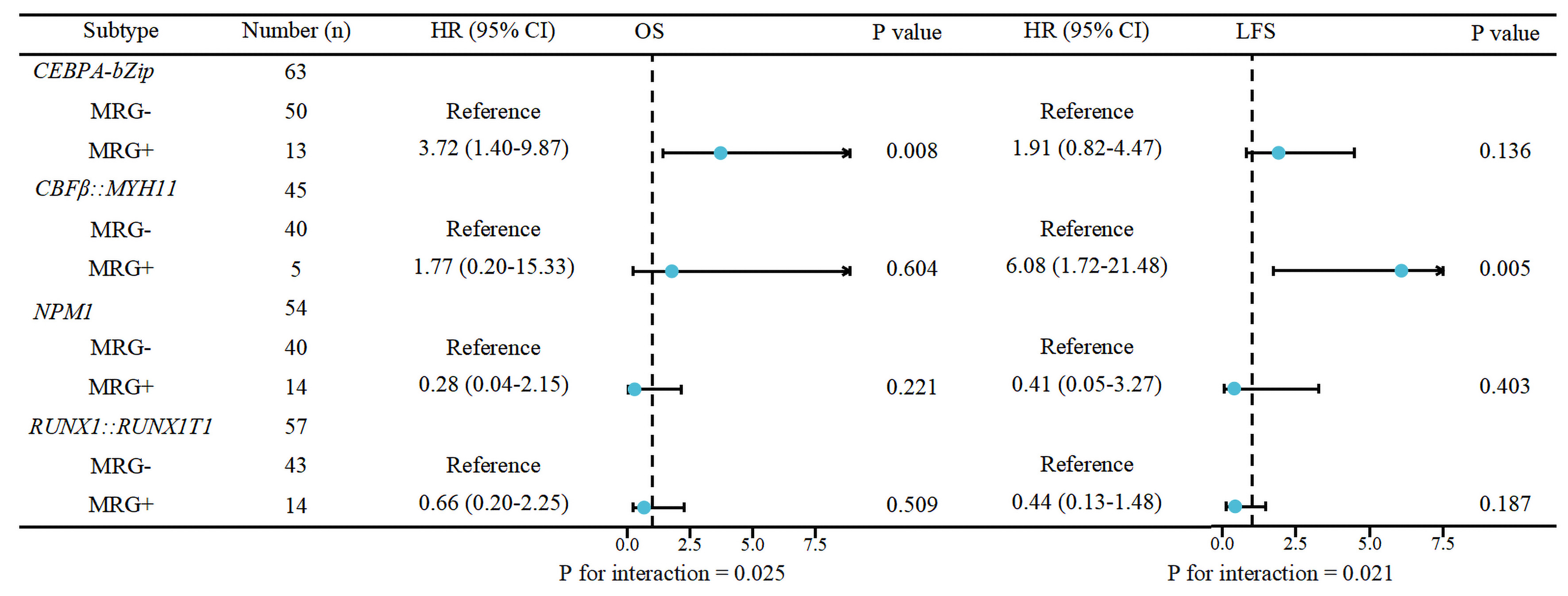

Supplement: IANN-2025-5157.R2-Supplementart Table -Clean copy.docx [file IANN_A_2636337_SM1569.docx]
